# Pharmacological Effects of *Houttuynia cordata* Thunb (*H. cordata*): A Comprehensive Review

**DOI:** 10.3390/ph15091079

**Published:** 2022-08-29

**Authors:** Shahzad Rafiq, Haihong Hao, Muhammad Ijaz, Ahmed Raza

**Affiliations:** 1 National Reference Laboratory of Veterinary Drug Residues, Huazhong Agricultural University, Wuhan 430070, China; 2 MOA Laboratory for Risk Assessment of Quality and Safety of Livestock and Poultry Products, Huazhong Agricultural University, Wuhan 430070, China; 3 Shenzhen Institute of Nutrition and Health, Huazhong Agricultural University, Shenzhen 518000, China; 4 Shenzhen Branch, Guangdong Laboratory for Lingnan Modern Agriculture, Genome Analysis Laboratory of the Ministry of Agriculture, Agricultural Genomics Institute at Shenzhen, Chinese Academy of Agricultural Sciences, Shenzhen 518000, China; 5 Department of Veterinary Medicine, University of Veterinary and Animal Sciences, Lahore 54000, Pakistan

**Keywords:** *Houttuynia cordata* Thunb, anti-inflammatory, anti-viral, anti-bacterial, immunomodulatory, anti-tumor

## Abstract

*Houttuynia cordata* Thunb (*H. cordata*) is a rhizomatous, herbaceous, and perennial plant widely distributed in Asia. It has multiple chemical constituents, such as alkaloids, essential oils, phenolic acids, and flavonoids used against various health problems. The essential oils and flavonoids are the main components of *H. cordata* that play an essential role in disease treatment and traditional health care. Moreover, the leaves and stems of *H. cordata* have a long medicinal history in China. In addition, *H. cordata* is used against several health issues, such as cold, cough, fever, pneumonia, mumps, and tumors, due to its anti-inflammatory, anti-bacterial, anti-viral, anti-oxidant, and anti-tumor effects. It protects organs due to its anti-inflammatory activity. *H. cordata* regulates immunity by enhancing immune barriers of the oral cavity, vagina, and gastrointestinal tract, and shows broad-spectrum activity against liver, lung, breast, and colon tumors. However, there are some gaps to be filled to understand its pathways and mechanisms. Mechanisms such as its interaction with cells, cell membranes, and various drugs are important. Studies in relation to the blood–brain barrier, lipophilicity, cAMP signaling, and skin permeability, including pharmaceutical effects, will be very useful. This review includes the biological and pharmacological activities of *H. cordata* based on up-to-date research.

## 1. Introduction

Eastern countries have a long history of using herbal medicine; in particular, Chinese people have used herbal medicine to treat various diseases for more than 8000 years [1]. The chemical components and biological activities present in the medicinal plant can be used for the prevention and treatment of various diseases [2]. Natural products with a variety of pharmacological targets in numerous diseases have emerged as crucial sources for the development of novel drugs [3]. The most popular anti-malarial drug, artemisinin, which is obtained from plants, is essential for the treatment of malaria [4]. The World Health Organization summarized data in 1985 that showed that, of the world’s total population, eighty percent of people depend on traditional medicine as a treatment, including herbal medicine [5].

*Houttuynia Cordata Thunb* (*H. cordata*) is a rhizomatous, herbaceous, and perennial plant found in China, Japan, Korea, and Southwest Asia. It usually grows in shady and moist places. This plant is widely distributed in China, and is present in Tibet, Gansu, Shaanxi, Yunnan, and eastern Taiwan [6]. As its aboveground stems and leaves have a long medicinal history in China and are employed in the treatment of pneumonia and lung abscesses, it plays a significant role in traditional health care and disease treatment [7].

In the past, people used *H. cordata* as medicine. In India, it is harvested as a medicine and daily food [8]. A significant amount of scientific evidence shows that *H. cordata*, as a whole plant or in the form of its extracts, has important medicinal effects [9]. Recently, several studies showed and unveiled its anti-allergic [10,11], anti-inflammatory [12,13], anti-viral [14], anti-oxidative [15], anti-leukemic [16], anti-cancer [17], and anti-SARS [18] activities. During the outbreak of severe acute respiratory syndrome (SARS), *H. cordata* was used for the prevention of SARS by the Health Ministry of China. It also helps to decrease swelling, lower fever, and drain pus, and in urination. Moreover, *H. cordata* also shows efficacy against methicillin-resistant *Staphylococcus aureus* (MRSA) [19] and multi-drug resistant *Escherichia coli* [20].

It is used to treat dysentery, fever, cold, and mumps in clinical medicine. It is also used to protect organs by reducing the release of inflammatory mediators. It boosts immunity by enhancing the immune barriers of the vagina, intestines, and other organs [7]. In this review, we briefly discuss the chemical properties of *H. cordata* and various effects on different organs of the body.

## 2. Chemical Components of *H. cordata*

*H. cordata* has a variety of chemical constituents with characteristic medicinal properties and belonging to different chemical groups, such as alkaloids, essential oils, and flavonoids as shown in Table 1 [21]. The alkaloids consist of aristolactam A, 3,4-dimethoxy-N-methyl aristolactam, lysicamine, noraritolodione, norcepharadione B, 3,5-didecanoyl-pyridine, 7-chloro-6-demethyl-cepharadione B, cis-N-(4-Hydroxystyryl) benzamide and trans-N-(4-Hydroxystyryl) benzamide, 2-nonyl-5-decanoylpyridine, 3,5-Didecanoyl-4-nonyl-1,4-dihydropyridine, cepharadione B, splendidine, piperolactam A, 3-decanoyl-4-nonyl-5-dodecanoyl-1,4-dihydropyridine, aristolactam B, 3,5-didodecanoyl-4-nonyl-1,4-dihydropyridine, 7-chloro-6-demethylcepharadione B, 3-nonylpyrazole, N-methyl-5-methoxy-pyrrolidin-2-one, phenanthrolactam compounds [22,23]. The flavonoid compounds included quercetin, rutin, hyperin, afzelin, quercitrin, isoquercitrin, kaempferol, quercetin hexoside, avicularin, apigenin, isorhamnetin, phloridzin, quercetin-3-*O-β*-D-galactoside-7-*O-β*-D-glucoside, and polyphenols include chlorogenic acid, vanillic acid, protocatechuic acid, catechin, *p*-hydroxy-benzoic acid methyl ester, chlorogenic acid methyl ester, cryptochlorogenic acid, neochlorogenic acid, procyanidin B, quinic acid, caffeic acid, *cis*-methyl ferulate, *trans*-methyl ferulate, methyl vanillate, vanillin, houttuynamide A, and houttuynoside A [24,25,26]. The main components of the essential oil are houttuynin, decanal, trans-caryophyllene, decanoic acid, camphene, β-pinene, lauraldehyde, α-pinene, limonene, nonanol and linalool bornyl acetate, methyl n-nonyl ketones, beta myrcene, monoterpene, 4-terpineol, caryophyllene oxide, phenylpropene derivatives, sesquiterpenes, and oxidized diterpenes [27].

*H. cordata* has many components, and alkaloids are abundant ingredients [28]. Essential oil and flavonoids are known to be major components that exert pharmacological activities. Moreover, decanoyl acetaldehyde in *H. cordata* has a fishy smell called Yu-Xing-Cao, and is a herb in traditional Chinese medicine [22]. It has anti-bacterial effects and is easily transformed to 2-undecanone at higher temperatures [29]. Steam distillation extracts of *H. cordata* contain some important oils, which consist of oxidized diterpenes, monoterpenes, sesquiterpenes, and oxidized diterpenes [30]. Others present in *H. cordata* include bornyl acetate (0.4–8.61%), ketones (2.10–40.36%), and β-myrcene (2.58–18.47%) [27]. Eleven ingredients have been isolated from leaves of *H. cordata,* and seven have been isolated from the roots and are not present in the leaves [7]. It is also reported that *H. cordata* from various areas has various anti-bacterial effects [31]. Flavonoids in *H. cordata*, such as quercetin, quercitrin, and hyperoside, are mostly combined with rhamnose in glycosides [26]. A new form of hyperoside and houttuynia has been isolated from flavonoid compounds in *H. cordata* [32]. Other new components are houttuynamide A and houttuynoside A [25]. Caffeic acid derivatives, quinic acid derivatives, chlorogenic acid, neochlorogenic acid, and cryptochlorogenic acid are considered the essential components of *H. cordata* [24]. Alkaloids such as phenanthrolactam, piperolactam, and aristololactam are key components of *H. cordata* and play an essential role in pharmacological effects [23].

**Table 1:** Therapeutic effect of different extracts of *H. cordata*.

Class	Compound	Chemical Structure	Therapeutic Properties	References
Essential Oil (Oil)	Terpenoids	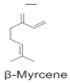	Anti-bacterial activities, anti-viral activities, and anti-inflammatory activities.	[12,33,34]
	Hydrocarbons	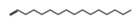 1-Hexadecene	Anti-bacterial activities, anti-viral activities, and anti-inflammatory activities.	[12,30,33]
	Esters	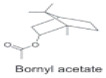	Anti-bacterial activities and anti--inflammatory activities.	[12,33]
	Alcohols	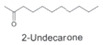	Anti-viral activities and anti-inflammatory activities.	[12,30,33]
Flavonoids	Quercetin	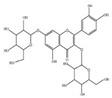	Inflammatory mediator, anti-viral	[9,35]
	Quercitrin	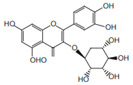	Anti-inflamatory	[36]
	Hyperin	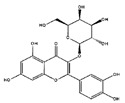	Anti-viral, anti-inflamatory	[9,37]
	Rutin	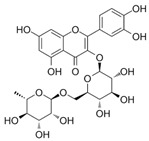	Inhibition of cholestasis	[9,38]
	Afzelin	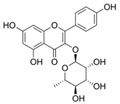	Lower inflamatory cytokines	[28,39]
	Kaempferol	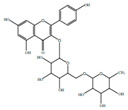	Anti-inflamatory	[9]
	Isoquercitrin	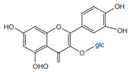	Anti-inflamatory	[9]
Alkaloids (Alkaloids)	Aristolactam A	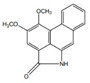	Anti-tumor activities	[9,17]
	Aristolactam B	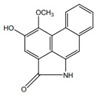	Anti-inflamatory	[9,23]
	Piperolactam A	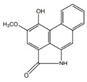	Anti-bacterial, anti-pyretic, detoxicant, anti-ulcer	[9,17]
	Lysicamine	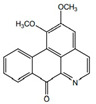	Anti-parasitic, anti-bacterial	[9,40]
	Norcepharadione B	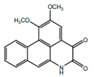	Anti-inflamatory, anti-pyretic	[9,17]
	3,4-Dimethoxy-N-methyl aristolactam	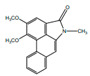	Anti-inflamatory	[9]
	cis-N-(4-Hydroxystyryl) benzamide	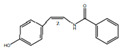	Inhibitor of platelet aggregation	[9,41].
	3,5-Didecanoyl-4-nonyl-1, 4-dihydropyridine	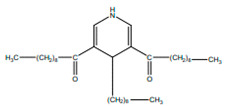	Anti-inflamatory	[9,21]
	7-chloro-6- demethylcepharadione B	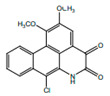	Anti-oxidant	[9]
	trans-N-(4-Hydroxystyryl) benzamide	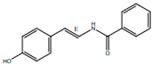	Inhibitor of platelet aggregation	[41]
	Cepharadione B	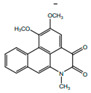	Anti-oxidant	[9]
	Splendidine	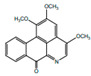	Inhibitor of platelet aggregation	[9,42]
Organic Acid	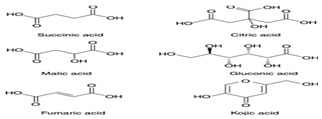		Anti-bacterial, anti-fungal activities	[43]
Polyphenols	Houttuynamide A	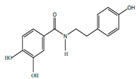	Anti-inflamatory	[9]
	Houttuynaside A	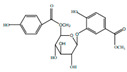	Anti-inflamatory	[9]
	Chlorogenic acid	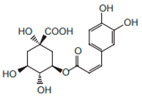	Anti-hypertensive	[9]
	Vanillin	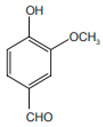	Anti-oxidant	[9]

## 3. Anti-Inflammatory Effects and Immunomodulatory Activity of *H. cordata*

Inflammation is a protective response of the body against offending agents such as viruses, bacteria, toxic chemicals, and damaged cells. There are two forms of inflammation. One is acute inflammation and the other is chronic inflammation [44]. Many cells are involved in inflammation, including neutrophils, eosinophils, basophils, mast cells, fibroblasts, and mononuclear cells [45]. Different extracts of *H. cordata*, such as water extract, n-butanol extract, and ethanol extract, have shown anti-inflammatory results in mice as shown in Table 2. All extracts have good anti-inflammatory activity. It has been found that the anti-inflammatory effects of water extract are better than those of ethnolic extract. Fresh *H. cordata* extracts showed better pharmacological activity than dry *H. cordata* extract [9].

Cells involved in body immunity are basophils, eosinophils, mast cells, lymphocytes, and neutrophils. They play vital immune functions. Antibodies, tumor necrosis factors, interferons, and interleukin also play an essential role in body immunity. Abnormal immune functions result in microcirculation, anaphylactic shock, and central nervous system disorders [46]. The polyphenols present in *H. cordata* show anti-allergic effects. Due to *H. cordata* extracts, decreased activity of iIgE and FcεRI expression on basophilic cells was observed. Moreover, mRNA activity associated with γ-chains and FcεRI was decreased, and histamine secretions were inhibited [47]. It was observed that *H. cordata* extract decreased cutaneous anaphylaxis in vivo in mice. The level of cAMP present in mast cells is enhanced by using *H. cordata,* which shows that *H. cordata* can speed up the recovery from allergic reactions. HCP-2 polysaccharides extracted from *H. cordata* regulate the expression of T cells with a dosage of 0.1–25 μg/mL. It increases tumor necrosis factor-α (TNF-α), immune molecule interleukin-1β, and macrophage inhibitory protein-1α and -1β, which increases body immunity. It has been recorded that *H. cordata* reduces Th2-mediated immune disorders. Ethanol extract of *H. cordata* decreases the migration of T cells, which ultimately strengthens immune response [10]. *H. cordata* extract helps in the regulation of immune mediators. After 18 h of treating vaginal epithelial cells with *H. cordata*, levels of leukocyte protease inhibitor mRNA and human β-defensin 2 were increased. Moreover, an increase in IL-2 and IL-6 and a decrease in CCL5 were observed. These findings show an increase in the overall immune response. *H. cordata* has the same effects on oral immune mediators by expressing human β-defensin 2, IL-8, CCL20, and secretory leukocyte protease inhibitor. In this manner, *H. cordata* regulates oral immune response [48].

### 3.1. In Vitro Anti-Inflamatory Effect of H. cordata

The water extract was reported to reduce nitric oxide and TNF-α production (by up to 30%) in LPS-induced mice macrophages at a dose rate of 0.06 and 0.12 mg/mL [13]. Additionally, it was discovered that the ethnolic extract of *H. cordata* inhibited the inflammatory biomarkers interleukin-6 and nitric oxide in lung epithelial cells (A549) and alveolar macrophages challenged with LPS (MH-S) [49]. In LPS-treated alveolar macrophages (MH-S) and lung epithelial cells, the ethanolic extract of *H. cordata* was observed to suppress inflammatory mediators such as interleukin-6 and nitric oxide [35]. In RAW 264.7 cells, essential oils extracted from HC considerably decreased the LPS-induced inflammation. The HC was withdrawn through supercritical CO_2_ with the concentration of one percent solution, and was found to decrease the PGE2 and nitric oxide levels by 80.4 percent and 98 percent, respectively, in RAW cells treated with LPS [50].

In LPS-primed RAW 264.7 cells, the active biomolecules sodium houttuyfonate and 2-undecanone that were extracted from *H. cordata* essential oil were demonstrated to have a significant anti-inflammatory impact. At the same concentration, sodium houttuyfonate showed a significant decrease in TNF-α (*p* < 0.001) and IL-1β level compared to 2-undecanone [29]. One of the crucial molecular pathways thought to be involved in the inflammatory pathophysiology associated with allergy is the mast cell-mediated anaphylactic shock. The primary causes of this condition are alterations in intercellular calcium ions (Ca^2+^) and histamine produced from degranulated mast cells that cause such severe anaphylactic responses. Research revealed the HCWE’s anti-allergic effects on both systemic rat peritoneal mast cells and acute anaphylactic responses (RPMCs). Histamine release and Ca^2+^ uptake have been demonstrated to be inhibited by the HCWE in a dose-dependent manner at concentrations of 0, 2.5, 25, and 250 µg [10]. The increase in adenylate cyclase activity and the subsequent rise in the intracellular cAMP level in mast cells was suggested to be the probable mechanism causing the suppression of histamine release and decreased Ca^2+^ absorption. Additionally, it was shown that IgE-mediated allergic reactions play a significant part in the pathophysiology of inflammation. The Fc epsilon RI receptor, a high-affinity IgR receptor expressed in human basophilic cells, has the ability to trigger allergic responses. Shim et al. demonstrated that the HC water extract decreased both the mRNA expression of both the chains of the Fc epsilon RI receptor in human KU812F cells and the IgE binding activity. Additionally, it was discovered that the HC extract inhibited KU812F cells’ Fc epsilon RI-mediated production of histamine [19].

A study was conducted by isolating polysaccrides from *H. cordata* and its composition was found to consist of galacturonic acid, glucose, galactose, rhamnose, and arabinose. It was found that *H. cordata* polysaccharide (HBHP-3) is an active ingredient that inhibits the expression of mRNA, and is involved in inflammatory activity and the secretion of nitric oxide, hence lowering inflammation in a dose-dependent manner [51]. Methanolic and aqueous extract of *H. cordata* were displayed anti-inflammatory properties. The protein denaturation method was used to assess the plant’s anti-inflammatory potential. Different levels of Houttuynia cordata free radical scavenging and anti-inflammatory activity are present in methanolic and aqueous extracts. The Houttuynia cordata methanolic extract was the most active of the extracts tested in the study compared to the aqueous extract [52].

**Figure 1:**
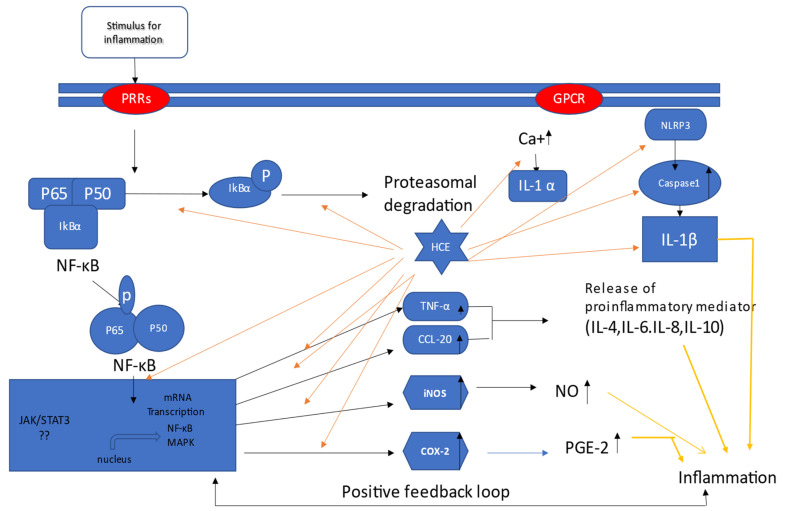
Mechanism of *H. cordata* acts as an anti-inflammatory agent in vitro; *H. cordata* suppresses inflammation through various pathways. It blocks all mentioned signaling pathways, resulting in a decrease in the production of inflammatory mediators such as prostaglandins and interleukins. Moreover, *H. cordata* suppresses inflammation through down-regulation of NF-κB signals and subsequent COX2, INOS, CCL-20, and TNF- α pathways [7].

These studies have provided ample evidence that *H. cordata* has anti-inflammatory capabilities against a variety of inflammagens in various cell lines. Based on the research, Figure 1 depicts a schematic mechanism via which *H. cordata* functions in several in vitro models.

### 3.2. In Vivo Anti-Inflamatory Effect of H. cordata

Ethanolic extract of the aerial parts of *H. cordata* was found to have anti-inflammatory effects on LPS in male ICR models of mice. Researchers observed that dosages of 100 and 400 milligrams per kilogram (mg/kg) reduced the total cell count by 46.1% and 66.5%, receptively. It was found to be as efficacious as dexamethasone, with a dose rate of 30 mg/kg [35]. Furthermore, when evaluated against a carrageenan-air pouch model, oral use of *H. cordata* supercritical extract at a 200 mg/kg dose suppressed albumin leakage, exudation, and infiltration of inflammatory cells in the ICR mice model. When the efficacy of this extract was compared with that of dexamethasone (2 mg/kg intraperitoneal) and indomethacin (2 mg/kg intraperitoneal), the extracts were found to be more effective at reducing both the cyclooxygenase 2/PGE2 and TNF-α/NO pathways [50]. In LPS-induced male ICR mice, quercitrin showed the greatest reduction in the cell count in the bronchoalveolar fluid after oral treatment of isolated flavonoids (hyperoside and quercitrin and afzelin at a dose rate of 100 mg/kg body weight) [28].

In a mouse model of D-galactosamine/LPS induction, the afzelin extracted from a methanolic fraction of HC lowered the quantity of inflammatory cytokines (TNF-α and IL-6) and AMPK expression, and enhanced the expression of Sirtuin-1 (Sirt-1) [39].

The effectiveness and potential mechanisms of the *Houttuynia cordata* (HC) extract in the treatment of (IC/BPS) interstitial cystitis/bladder pain syndrome were studied. A total of 30 adult female rats were divided randomly into three groups: a sham group (n = 10), a cyclophosphamide + saline (CYP + NS) group (n = 10), and a cyclophosphamide and Houttuynia cordata extract (CYP + HC) group (n = 10). The IC/BPS animal model was induced with the CYP + NS group and the CYP + HC group received cyclophosphamide (75 mg/kg, intraperitoneal injection, once every three days for 10 days), and the sham group rats received an equal volume of saline injection. Then, the three groups’ differences in urine frequency, nociceptive behaviours, cystometry, bladder weight, histological alterations, and cytokine (IL-6, IL-8, and TNF- α) concentration were assessed. The CYP + NS group considerably outperformed the sham and CYP + HC groups in terms of inflammatory grade, mast cell number, fraction of activated mast cells, bladder weight, cytokine concentration of bladder homogenates, and frequency of urination. Additionally, compared to the CYP + NS group, the CYP + HC group had a longer intercontraction interval, a larger bladder capacity, a higher nociceptive threshold, fewer mast cells, and a lower proportion of activated mast cells (*p* < 0.01) [53].

The second-phase anti-edematous effect of 3.08 and 6.16 mL/kg HCFP, which is comparable to that of diclofenac (150 mg/kg), was shown to diminish paw oedema in Wistar rats after 2 h of carrageenan stimulation. By comparison, 6.16 mL/kg HCFP also decreased paw oedema after 1 h of carrageenan stimulation, indicating the first-phase anti-edematous impact. Syringic, vanillic, p-hydroxybenzoic, and ferulic acids were among the active phenolic compounds quantified by HPLC that had anti-inflammatory properties [54]. These studies showed the anti-inflammatory property of *H. cordata* in vivo.

**Table 2:** Different extracts of *H. cordata* to treat inflammation in vitro and vivo.

Extracts	Doses	Standard Drug	Inflammagen Used	Model Used	Time Period	Minimal Active Concentration	Most Potent Biomolecule	Reference
Aqueous	0.5–3 g/kg	None	DNA-BSA	ICR mouse	2 h	0.5 g/kg	Not mentioned	[55]
Aqueous	1, 10, 20 µg/mL	None	DNA-BSA	RBL-2H3	30 min	1 µg/mL	Not mentioned	[55]
Aqueous	1, 2 g/mL	None	Acetaminophen	BALB/cA mice	4 weeks	2 g/L	Not mentioned	[29]
Ethanol (100%)	0.1, 0.2, 1%	None	LTA (1 µg/mL)	RT-7	24 h	0.5%	Not mentioned	[19]
Ethanol(80%)	400, 600, 100 mg/kg	None	Oxaliplatin	Male Sprague Dawley rat	15 days	1000 mg/kg/day	Not mentioned	[56]
Ethanol	0.05, 0.1, 0.2 mg/mL	None	PMA + Ca^2+^ ionophore	HMC-1	5 h	0.2 mg/mL	Not mentioned	[57]
Essential oil	0.01, 0.1, 1, 10, 100 µg/mL	NS-398	LPS (1 µg/mL)	Mouse peritoneal macrophange	24 h	100 µg/mL	Not mentioned	[58]
Essential oil (sodium houttuyfonate and two undecanone)	0.1,1,10,20 µg/mL	None	LPS (1 µg/mL)	RAW 264.7	24 h	1 µg/mL	Sodium houttuyfonate	[29]
Essential oil (sodium houttuyfonate and 2 undecanone)	100, 200, 400 mg/kg	Aspirin	Xylene	Mouse	30 min	200 mg/kg	Sodium houttuyfonate	[29]
Polysaccrides	40, 80, 160 mg/kg	Dexamethasone	LPS	BALB/cA mice	24 h	40 mg/kg	Not mentioned	[59]
Essential oil	20, 40 mg/kg	Dexamethasone	Xylene, Formaldehyde	Mouse	7 days	20 mg/kg	2-undecane, n-Decanoic acid, Hexadecanoic acid-methyl ester,	[33]

## 4. Effect of *H. cordata* on Different Organs

Lung inflammation is one of the most important signs during lung infection. *H. cordata* has an anti-inflammatory property that plays a significant role in treating lung inflammation. Quercetin obtained from HC, when administered orally at a dose rate of 100 mg/mL in an LPS-induced model, significantly decreased the production of NO and inflammatory mediators such as cytokines [35]. Researchers compared the effect of different dosage levels of flavonoid glucoside extract of *H. cordata* at 50, 100, and 200 mg/kg compared with ribavirin 100 mg/kg with the use of acute injury of lung tissues by the H1N1 virus. At 14 days, they found a lower lung index and less weight loss [37]. The oxidative lung damage caused pulmonary fibrosis. In rats, when pulmonary fibrosis was induced by bleomycin, *H. cordata* aqueous extracts showed a better and stronger anti-oxidant property than vitamin E by decreasing concentration of hydroxyproline, superoxide dismutase, and malondialdehyde [60].

The intestinal barrier is a structure that allows uptake of essential nutrients, while restricting pathogenic molecules and bacteria. The microflora present in the intestine also play a vital role in protecting the intestine [61]. The constituents of *H. cordata* are polysaccharides, and sodium houttuyfonate is instrumental in reducing or regulating the production of mucus from the goblet cells and wart formation of Secretory IgA (antibodies in the secretions and excretions). Moreover, the protein ZO-1, which forms a gap junction between the intestinal cells, is up-regulated or enhanced to compact intestinal, mechanical, and immunological barriers [62]. Intestinal inflammation induced by *Salmonella typhimurium* is dampened by sodium houttuyfonate in the form of up-regulating tight junction proteins between the mucosal cells of the intestine and the signaling pathway that leads to interleukin production [63]. These studies showed that *H. cordata* has a therapeutic effect on the GIT system.

Oxidative damage and inflammation disrupt salt metabolism, and the body’s water is a common cause of kidney diseases [64]. Treatment with 1 to 2% water extracts of *H. cordata* lowered creatine kinase activity and urea nitrogen in diabetic mice having kidney injury [65]. Inflammation can also cause serious kidney damage. A study found that sodium houttuyfonate dramatically decreased the expression of MCP-1 and NF-kB at a dose rate of 60 to 120 mg/kg in a dose-dependent manner [66]. These findings suggest that *H. cordata* extract is helpful in various kidney diseases.

Chemical compounds such as sodium houttuyfonate, flavonoids, and polysaccharides present in *H. cordata* have been used for the treatment of pneumonitis. *H. cordata*, a pro-inflammatory cytokine (IL 6) and IL 6, were reduced through oral use of quercetin (flavonoid) extracted from *H. cordata* at a dose rate of 100 mg/mL [35]. It was found that sodium houttuyfonate extracted from *H. cordata* causes a reduction in mRNA levels of MyD88, TLR4, and NF-κB p65 when used at a dose rate of 24.3 mg/kg. Macromolecular polysaccharides of *H. cordata* reduce lung injuries, pulmonary oedema, and pneumonia [67]. A combination of cell transplantation with *H. cordata* is used to treat lung inflammation. The extract at a dose rate of 50 mg/kg reduces the lungs’ inflammatory response. For infection caused by the H1N1 virus, extracts of flavonoid glycoside with a dose rate of 50, 100, and 200 mg/kg cause less weight loss and a lower lung index compared to the anti-viral drug ribavirin with a dose of 100 mg/kg. It causes inhibition of H1N1 neuraminidase activity, which reduces lung inflammation [37]. Water extract of *H. cordata* remarkably reduces inflammation of the lungs caused by bleomycin-induced pulmonary fibrosis in rats. It also has strong anti-oxidative activity compared to vitamin E. The active ingredients of *H. cordata* include polysaccharides, flavonoids, and sodium houttuyfonate, which have remarkable anti-inflammatory activity in the lungs [68].

*H. cordata* protects the intestine various barriers (mucosal barriers, chemical, mechanical, biological, and immune barriers) are present in the intestine. Moreover, intestinal flora also play an essential role in protecting the intestines. Recently, it has been found that sodium houttuyfonate and polysaccharides extracted from *H. cordata* decrease the expression of sIgA, intestinal goblet cells, and tight junction protein present in the intestines. Sodium houttuyfonate is also involved in reducing inflammation caused by *Salmonella typhimurium*. Regulation of bacteria, such as Vibrio and Bacillus, also includes polysaccharides made up of galactose, glucose, rhamnose, and arabinose at a 40 mg/kg dosage. These findings show that sodium houttuyfonate and polysaccharides of *H. cordata* have protective activity by inhibiting NF-κB and regulating intestinal flora in the intestines [69].

Many natural extracts from plants have effective results in preventing and treating various liver ailments. For instance, the chemical components of extracts such as terpenoids, glycosides, coumarins, and alkaloids prevent liver fibrosis. Cholestasis is a common problem inhibited by compounds such as quercetin and rutin. Liver cells are very sensitive to oxidative stress. *H. cordata* ethyl acetate extract reduces liver damage through its anti-oxidant activity. Ethyl acetate of *H. cordata* extract at a dose rate of 1000 mg/kg causes an increase in superoxide dismutase, glutathione, and catalase enzymes, and a decrease in malondialdehyde and serum transaminase resulting in liver protection. The mixture of ethanol and water extract of *H. cordata* at a dosage of 300 mg/kg/day for seven days reduces oxidative factors in the liver [38].

Oxidative damage, inflammation, and infections caused by various pathogenic organisms are the major factors involved in kidney problems. It was observed that 1 to 2% of *H. cordata* water extract reduced the level of serum creatinine and blood urea nitrogen and oxidative factors in the kidney. Moreover, 2% extract of *H. cordata* causes inhibition of membrane-anchored receptor made up of end products (RAGE) and glycation, which activate mitogen-activated protein kinase. They induce intracellular reactive oxygen species generation and are involved in renal protection. Sodium houttuyfonate present in *H. cordata* causes a decrease in expression of MCP-1 and nuclear NF-κB at a dosage of 60–120 mg/kg. It protects against renal glomerulonephritis and kidney oxidative stress [66].

Anti-oxidants such as catechin and procyanidin B present in *H. cordata* intervene in remodeling of the heart. The use of 2% *H. cordata* water extract was found to down-regulate cardiac activity related to oxygen, interleukin-6, inflammatory factors, and protein carbonyl. Moreover, 1 and 2% of *H. cordata* water extract block expression of NF-κB p65, p47phox, and p-p38 in the mouse heart. Sodium houttuyfonate shows activity against myocardial hypertrophy induced by isoprenaline with a dosage of 90 and 180 mg/kg up to 1 week. Cyclic adenosine, left ventricular weight index, heart weight index, and angiotensin 2 were also decreased using sodium houttuyfonate. Moreover, the cross-sectional area of cardiomyocytes and expression of hydroxyproline was also reduced [70]. Sodium houttuyfonate with 50 and 100 mg/kg dosages causes down-regulation of renin-angiotensin-aldosterone, which involves controlling blood pressure. Sodium houttuyfonate is also associated with NF-κB pathway inhibition and adenosine monophosphate-activated protein kinase at the same dosage. It also reduces heart fibrosis and myocardial inflammatory factors. *H. cordata* reduces the release of inflammatory mediators of the heart and oxidative damage to the heart. Sodium houttuyfonate present in *H. cordata* also affects the sympathetic nervous system and the renin-angiotensin system by reversing hypertrophy and remodeling of myocardium. Sodium houttuyfonate treatment elevated the activation of adenosine monophosphate-activated protein kinase (AMPK) on post-infarct heart and post-hypoxia H9C2. AMPK did not suppress NF-κB signaling directly; its inhibition of NF-κB was realized indirectly via its downstream mediators, e.g., Sirtuin-1 (SIRT1), Forkhead box O (F oxO) family, and peroxisome proliferator-activated receptor γ co-activator 1α (PGC-1α). Therefore, AMPK activation and suppression of NF-κB and inflammatory cytokines was critically involved in the anti-remodeling effect of SH post-myocardial infarction. [71].

## 5. Anti-Oxidant Effects of Various Extracts of *H. cordata*

In this study, anti-oxidant measurement tools for *H. cordata* were 1,1-Diphenyl-2-picrylhydrazyl (DPPH), 2,2′-azinobis (3-ethylbenzothiazoline-6-sulfonic acid) diammonium salt (ABTS), and β carotene assays, as shown in Table 2. IC50 values are defined as various concentrations of samples needed to scavenge 50% DPPH and ABTS free radicles present in test solutions. The most effective scavenger solution was methanol extract of *H. cordata,* and the second ranked was ethanol extract. However, there was no significant difference between IC50 values of ethanol and methanol extracts. Standard BHT was present in ABTS assay (*p <* 0.05). Chloroform and hexane extracts of *H. cordata* were weak radical scavengers in ABTS and DPPH models (Table 3).

In the beta-carotene assay, the lipid peroxidation inhibition (LPI) with a data range of 63.35–86.61% was found in all extracts of *H. cordata* mentioned in Table 3. Higher LPI values indicate higher anti-oxidant activity. LPI methanol extract was found to have the highest values among all other extracts. Water and hexane extracts of *H. cordata* had the lowest anti-oxidant values [72]. The significant anti-oxidant effects of methanol extract of *H. cordata* are associated with many phenolic compounds [71].

## 6. Anti-Tumor Activity of *H. cordata*

**Figure 2:**
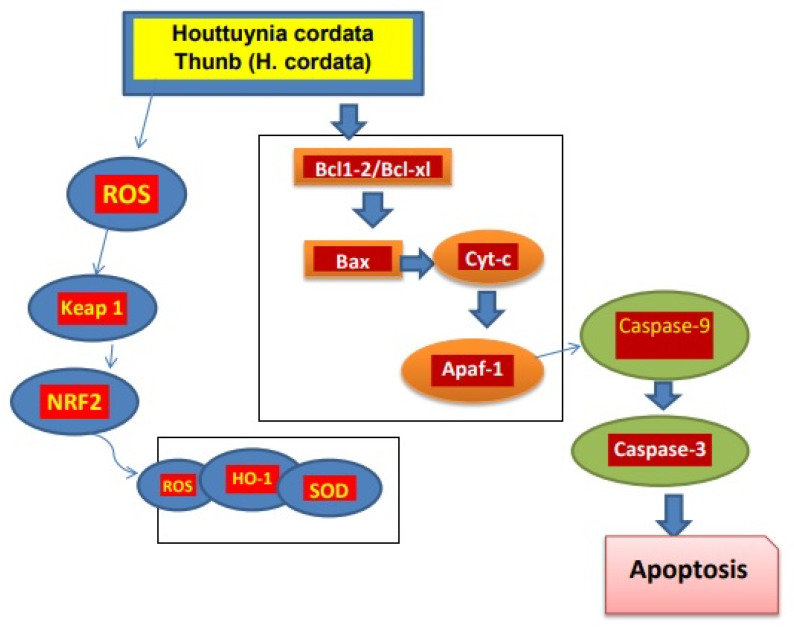
Mechanism of *H. cordata* acts as an anti-tumor agent. *H. cordata* suppresses cancerous cells by blocking NRF2/Bcl1, Bcl-xl signaling pathway and leading to apoptosis of cancerous cells [7].

In a study of mice with lung tumors induced by benzo-pyrene, it was found that the active components of *H. cordata*, such as 2-undecanone, had an anti-tumor effect that may be due to Nrf2-HO-1/NQO-1 pathway activation, which reduces inflammation of lung cells and damage of DNA. In addition, no signs of systemic toxicity were recorded [73]. Moreover, the polysaccharides present in *H. cordata* exhibited anti-tumor potential. The polysaccharide HCA4S1 inhibited proliferation of tumor cells by cancer cell cycle/A549 lung tumor arrest and apoptosis. Similarly, after HCA4S1 treatment, the activities of cyclin B1 and cleaved caspase3 in cells dramatically reduced [74]. The extracts of *H. cordata* with the concentration of 0 to 80 µg/mL caused a decrease in lipid accumulation in HepG2 cells when HepG2 cells were merged with a high level of glucose [75]. The ethanolic extract of *H. cordata* had anti-cancer effects against the colon cancer cell line HT-29. Cancer cell apoptosis was induced when treated with 450 µg/mL extract, which also resulted in lower mitochondrial membrane potential and increased reactive oxygen [76]. *H. cordata* also has activity against breast cancer. The development and progression of tumors are significantly influenced by the overexpression of the HER2/neu (receptors on breast cells) receptor. With an IC50 of 5.52 µg/mL, Houttuyninum suppressed HER2 phosphorylation in a dose-dependent manner in MDA-MB-453 cells without altering the expression of the HER2/neu protein. Additionally, HER2/neu-mediated signal transduction pathway downstream molecules ERK1/2 and AKT were blocked by houttuyninum from becoming activated [77]. At the concentration of 100 to 500 µg/mL, the ethanolic extract of *H. cordata* promotes apoptosis in breast cancer cells [78]. These studies showed that *H. cordata* has anti-tumor activity (Figure 2).

## 7. Effect of *H. cordata* on Viruses

The research on plants for the treatment of AIDS has made significant progress over the last ten years. Many plants and their products, such as *H. cordata,* have been found to have anti-HIV properties [79]. In vitro, the steam distillate and three main components from *H. cordata* manifested virucidal effects against HSIV-1 and influenza. The pretreatment with the distillate for 2 and 6 h, respectively, resulted in the inactivation of 20% and 40% of HIV-1 at two-fold dilution [80]. *H. cordata* aqueous extract has immunomodulatory and anti-SARS properties. *H. cordata* causes an increase in the spread of mouse splenic lymphocytes. According to flow cytometry, *H. cordata* enhanced the fraction of CD4+ and CD8+ T cells. Furthermore, it increased the interleukin 2 and interleukin 10 releases by mouse splenic cells. Regarding anti-viral activity, *H. cordata* inhibited the 3C-like protease of the SARSCOV and RNA-dependent RNA polymerase [81]

The flavonoid quercetin 7-rhamnoside (Q7R) present in *H. cordata* has anti-viral properties against the porcine epidemic diarrhea virus, the most common cause of severe enteropathogenic diarrhea as shown in Table 4. Using 0.014 μg/mL, it suppressed porcine epidemic diarrhea virus (PEDV) replication by 50% [82]. When tested for neuraminidase activity, *H. cordata* had anti-viral activity against the influenza virus and it totally suppressed viral neuraminidase with a dosage of 250 mg of herb/mL [83]. The flavonoids of *H. cordata* had anti-viral activity against the virus H1N1 both in vivo and in vitro. The combined action of hyperoside, rutin, quercitrin, and isoquercitrin in the extract significantly improved the life span and survival rate of mice suffering from H1N1. *H. cordata* extracts with a dose rate of 50, 100, and 200 mg/mL decreased H1N1 virus effects in lung tissues and decreased neuraminidase action of the virus [37]. Herpes simplex virus (HSV) infection was efficiently suppressed by *H. cordata* hot water extract, which might be due to the stoppage of the NF-κB pathway [84]. *H. cordata* inhibits avian infectious bronchitis virus, chicken embryo cells in vero cells and kidney cells, and decreases viral infection by up to 90 percent when detected with plaque reduction and reverse transcription PCR [85]. *H. cordata* water extract has anti-viral efficacy against the dengue virus serotype 2 strains 16,681. After pre and post-incubation with HepG2 cells, *H. cordata* at the dose rate of 10 to 100 mg/mL showed a significant reduction in intracellular DEN2 RNA synthesis, corresponding to a drop in dengue protein expression [84].

Coronaviruses are found throughout the world and infect humans and other animals. There are now seven types of coronaviruses that can infect humans. Recently found, severe acute respiratory syndrome coronavirus 2 (SARSCOV2) is one of these, and has caused millions of fatalities globally [86]. Three important proteins of SARS-CoV-2 are papain-like protease, protease (M^pro^), and ADP ribose phosphatase. Molecular docking of LigPrep, Epic, and Glide modules of the Schrödinger suite 2020–3 indicated that phytocompound (ligand) 6-hydroxyondansetron had an affinity for PLpro and (M^pro^) receptors. Results suggest that *H. cordata* may have therapeutic potential [87].

**Table 4:** Anti-viral effect of *H. cordata*.

Name of Extract	Name of Virus	Reference
Quercetin 7-rhamnoside (Q7R)	porcine epidemic diarrhea virus	[82]
Hyperoside, rutin, quercitrin	H1N1	[37]
Hot water extract	Herpes simplex virus	[84,88]
*H. cordata* water extract	dengue virus serotype 2 strains 16,681	[84]
*H. cordata* water extract	coronavirus 2 (SARSCOV2)	[84]
*H. cordata* polysaccharides	Murine Norovirus-1	[89]
Houttuynoid A	Herpes Simplex Virus type-1	[88]
Quercetin, quercetrin and cinanserin	Dengue fever virus, Corona virus	[90]
*H. cordata* extract	Enteric Virus	[91]
*H. cordata* essential oil	Avian infectious bronchitis	[92]

## 8. Anti-Bacterial Effect of *H. cordata*

Staphylococcus aureus is a food-borne, gram-positive bacterium that can cause infection of the skin, nasal cavity, GIT, and other human parts. MRSA was synergistically inhibited by sodium houttuyfonate and EDTA-Na_2_. Mice infected with MRSA were given sodium houttuyfonate combined with EDTA-Na_2._ After 28 days of MRSA infection, the survival rate of mice with sodium houttuyfonate treatment combined with EDTA-Na_2_ was 75 percent. It was significantly higher than the 43.75 and 50 percent survival rates of mice treated independently with EDTA-Na_2_ and sodium houttuyfonate, respectively [93]. At doses of 500 and 50 mg/mL, the aqueous *H. cordata* extracts demonstrated anti-bacterial effects against isolates of MDR *E. coli*, with the maximum and minimum zone diameters of inhibition of 29 and 13 mm, respectively. These findings suggest that *H. cordata* water extract (HCWE) has anti-microbial action against MDR *E. coli* in vitro [20]

*Pseudomonas aeruginosa* is a Gram-negative bacterium that infects deep wounds of the body and causes systemic illness. It was reported that sodium houttuyfonate had anti-bacterial activity against pseudomonas aeruginosa. The biosynthesis of alginate, a key ingredient for PA biofilm development, was suppressed, and is linked to sodium houttuyfonate’s down-regulation of algD and algR genes. Simultaneously, electron microscope observations showed that the bacteria’s shape changed after treatment, and the amount of alginate present in bacterial biofilm decreased [94].

Water extracts of *H. cordata* were found to have anti-bacterial effects against salmonellosis. It was observed that, after 8 h, the anti-bacterial activity of *H. cordata* increased with concentrations of 25 to 100 mg/mL. Bacterial absorption and morphologic alterations of body cells showed that there was no significant difference in the replication of bacteria. *H. cordata* showed a decrease in the pathogenicity of salmonella bacterium. The death rate of bacteria at the 7th day in the untreated group was 100%, and with a dose rate of 25, 50, and 100 μg/mL of *H. cordata,* the extract group lived up to 11, 17, and 23 days, respectively. It was recorded that *H. cordata* water extract is effective and safe in treating salmonella bacteria infections and various replicating pathogens [95].

## 9. Toxicity of *H. cordata*

*H. cordata* is an edible plant. Therefore, the toxic level of this plant is mostly ignored. However, it has been reported in some studies that aristolactams and aristolochic acid present in *H. cordata* can cause cancer [60].

Increased levels of aristolochic acid in liver cells also cause toxicity of proximal tubule epithelial cells present in the kidney. Aristolochic acid is also toxic in vivo because of its mutagenicity. A study revealed that 95% ethanol extracts from *H. cordata* show potential toxicity to zebrafish. A single dose of 2000 mg/kg of *H. cordata* with oral use had no harmful effects in mice during 14 days of treatment. However, oral administration of *H. cordata* with a dosage of 500–1000 mg/kg/day for 28 consecutive days led to some rats’ death. The histopathological examination showed inflammatory cell infiltration and vacuum degeneration of liver tissue, and focal necrosis of epithelial cells in kidneys. However, *H. cordata* has shown a very weak potential for toxicity. There is no evidence that *H. cordata* causes long-term toxicity. Nonetheless, *H. cordata* leaves and rhizomes are consumed in South China as an agricultural vegetable [96].

Research was conducted in 2018 to evaluate the toxicological effect of fermented Houttuynia cordata juice (FHJ) in a rodent model. FHJ was prepared by fermentation of *Houttuynia cordata* for 30 days and its active ingredients were evaluated. Due to lactic acid production, it has a lower pH of 3.63. Rats were fed with FHJ for 60 days and toxicological effects were evaluated using different biochemical, hematological, and histological tests. These revealed that there was no significant biochemical, histological, or hematological change in rats when compared with the control group. Therefore, it was postulated that FHJ did not have any toxicological effect in rats; hence, experiments should be conducted with humans in safety and toxicological studies. [97].

## 10. Conclusions

*H. cordata* is an herbal and medicinal plant with various medical and biological effects. It has various functions, such as protecting organs, and has anti-inflammatory effects concerning bacteria, viruses, and tumors, and anti-oxidant activities [98]. Aristolochia components from *H. cordata* show toxicity to kidneys, which is not considered beneficial for health. However, data about its toxicity is currently insufficient. Similarly, anti-liver activity and liver injury associated with *H. cordata* are unclear. Overall, *H. cordata* is considered beneficial in reducing inflammation of various internal organs. Water extract, flavonoids, sodium houttuyfonate, volatile oil, ethanol extract, and polysaccharide components of *H. cordata* inhibit the release of inflammatory mediators for heart remodeling, lung injury, and other pathological changes in tissues. Apoptosis is the most important function of *H. cordata* associated with liver, lung, gastric, colon, and breast cancer. *H. cordata* is also found to be used in eye drops for the treatment of vernal keratoconjunctivitis, and treatment of mild to moderate acne and non-inflammatory skin lesions. It is also used to cure insulin resistance associated with diabetes. However, based on previous studies, it was concluded that the components of *H. cordata* extracts are still unclear, and their effects are not fully characterized [99].

## 11. Future Aspects

Due to the different components of *H. cordata*, its various pharmacological effects seem too optimistic. There are some gaps to be filled in the future to provide and understanding of its pathways and mechanisms, as follows:

1. Mechanisms of *H. cordata*, such as interaction with cells, the cell membrane, and various drugs, will be of great importance.
2. Studies on its relation to the blood–brain barrier, lipophilicity, cAMP signaling, and skin permeability with pharmaceutical effects will be of utmost use. However, its possible side-effects and toxic effects should be studied carefully.
3. More data are required to study the pharmacological and toxicological activity of *H. cordata*.

## Figures and Tables

**Table 3:** Anti-oxidant effects of different extracts from *H. cordata*.

Activities of Different *H. cordata* Extracts	* DPPH IC50 (mg/mL)	* ABTS IC50 (mg/mL)	* LPI%
Hexane	NA	6.14 ± 0.67 a	67.43 ± 1.28 c
Chloroform	NA	4.64 ± 0.67 b	81.80 ± 0.91 b
Acetonitrile	NA	0.59 ± 0.01 d	83.69 ± 0.50 b
Ethanol	0.10 ± 0.01 b	0.42 ± 0.01 d	85.89 ± 0.82 b
Methanol	0.05 ± 0.00 c	0.23 ± 0.00 d	86.61 ± 0.76 ab
Water	0.22 ± 0.01 a	2.19 ± 0.03 c	63.35 ± 5.43 c

Values are means of three replications with standard deviations (SD). The lower IC50 value shows higher anti-oxidant activities in DPPH and ABTS assays. In the same column, means followed by different letters are significantly different (*p* < 0.05). Different letters a, b, c, d in the same column indicate that sample readings are statistically different from each other. Same letters in the same column indicate that sample readings are statistically significant from each other. Dibutyl hydroxytoluene (BHT) was used as a standard compound. NA: Not applicable [72]. * DPPH: 1,1-Diphenyl-2-picrylhydrazyl; * ABTS: 2,2′-azinobis (3-ethylbenzothiazoline-6-sulfonic acid) diammonium salt; * LPI %: Percentage of Lipid peroxidation inhibition.

## Data Availability

Data sharing not applicable.
